# Pre-surgical cryoablation in ≤ 2 cm ER + /HER2-tumors. Prognostic factors for the presence of residual invasive carcinoma

**DOI:** 10.1007/s10549-024-07325-7

**Published:** 2024-05-30

**Authors:** María José Roca Navarro, Jose Mª Oliver Goldaracena, Diego Garrido Alonso, Ylenia Navarro Monforte, Teresa Díaz de Bustamante Durbán, Mª Vicenta Córdoba Chicote, Fernando García Martínez, Covadonga Martí Álvarez, Laura Yébenes Gregorio, Jose Luis Montes Botella, Carmen Martín Hervás, José Ignacio Sánchez Méndez

**Affiliations:** 1 Autonomous University of Madrid, Madrid, Spain; 2 La Paz University Hospital, Paseo de La Castellana, 261, 28046 Madrid, Spain; 3 Rey Juan Carlos University, Madrid, Spain

**Keywords:** Breast cancer, Cryoablation, Ultrasound guidance, Infiltrating ductal carcinoma, Early stage, Low risk

## Abstract

**Background:**

Breast cancer remains the most commonly diagnosed cancer in women. Breast-conserving surgery (BCS) is the standard approach for small low-risk tumors. If the efficacy of cryoablation is demonstrated, it could provide a minimally invasive alternative to surgery.

**Purpose:**

To determine the success of ultrasound-guided cryoablation in achieving the absence of Residual Invasive Cancer (RIC) for patients with ER + /HER2- tumors ≤ 2cm and sonographically negative axillary nodes.

**Materials and Methods:**

This prospective study was carried out from April 2021 to June 2023, and involved 60 preoperative cryoablation procedures on ultrasound-visible, node-negative (cN0) infiltrating ductal carcinomas (IDC). Standard diagnostic imaging included mammography and tomosynthesis, supplemented by ultrasound-guided biopsy. MRI was performed in patients with associated intraductal carcinoma (DCIS) and an invasive component on core needle biopsy (18 out of 22 cases). All tumors were tagged with ferromagnetic seeds. A triple-phase protocol (freezing–thawing-freezing) with Argon was used, with an average procedure duration of 40 min.

A logistic regression model was applied to determine significant correlation between RIC and the study variables.

**Results:**

Fifty-nine women (mean age 63 ± 8 years) with sixty low-risk unifocal IDC underwent cryoablation prior to surgery. Pathological examination of lumpectomy specimens post-cryoablation revealed RIC in only one of 38 patients with pure IDC and in 4 of 22 mixed IDC/DCIS cases. All treated tumors had clear surgical margins, with no significant procedural complications.

**Conclusions:**

Cryoablation was effective in eradicating 97% of pure infiltrating ER + /HER2-tumors ≤ 2cm, demonstrating its potential as a surgical alternative in selected patients.

**Graphical Abstract:**

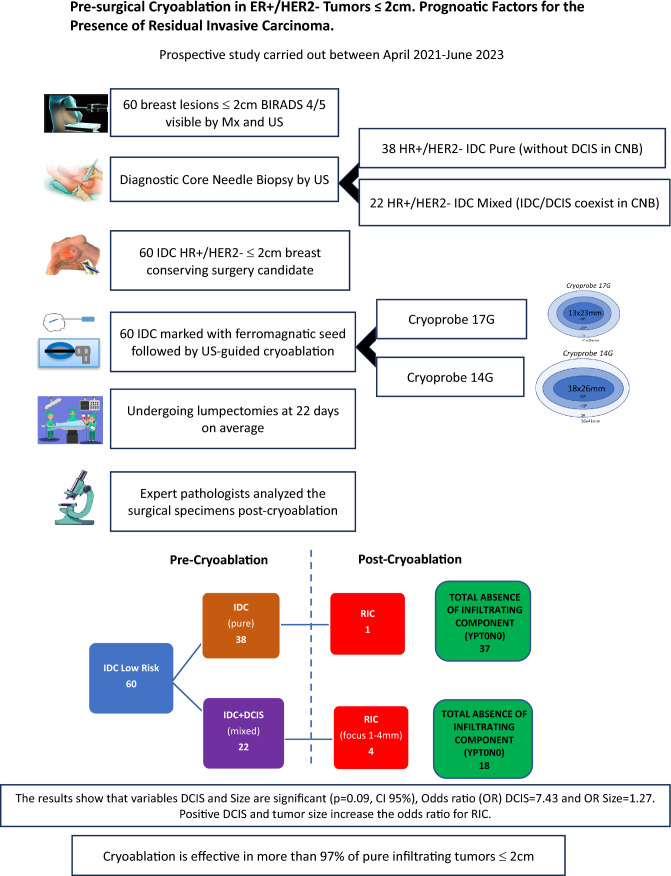

**Supplementary Information:**

The online version contains supplementary material available at 10.1007/s10549-024-07325-7.

## Introduction

Estrogen receptor-positive (ER +)/HER2-negative (HER2-) breast cancers measuring ≤ 2 cm typically exhibit a favorable prognosis due to their distinct molecular features and behavior, with minimal rates of recurrence and distant metastasis within 5 years [1]. According to the National Comprehensive Cancer Network (NCCN) guidelines, standard treatment is breast-conserving surgery (BCS), followed by adjuvant therapy, like radiotherapy and hormone therapy [2].

Percutaneous ablative techniques offer multiple advantages over conventional surgery. These treatments are performed using fine-gauge needles guided by imaging modalities such as ultrasound (US) or computed tomography (CT). Notably, these techniques achieve outstanding precision by administering substances or energy capable of obliterating tumors while preserving the surrounding tissue.

A variety of techniques utilize high temperatures for ablation, including radiofrequency, microwaves, and laser. Cryoablation is a different type of thermal ablation that exposes tissues to extreme cold, reaching temperatures of −40°. This method has gained approval from the American Society of Breast Surgeons and the Food and Drug Administration (FDA), for the treatment of fibroadenomas measuring less than 4 cm. In 2023 Diaz de Bustamante et al. published their experience in the management of fibroadenomas with cryoablation [3–5].

Cryoablation offers several advantages such as causing minimal damage to adjacent tissues, eliminating the need for sedation due to its intrinsic anesthetic properties.

Due to its ability to introduce intact or minimally degraded tumor antigens into the bloodstream, this technique may induce a host immune response against the tumor. This immunogenic effect under study could facilitate the regression of tumors outside the primary ablation zone, known as the abscopal effect [3, 6, 7].

When performing cryoablation using argon or nitrogen gas, cellular ultrastructure destruction occurs via the three-phase freezing–thawing protocol. In the first freezing phase, an increase in intracellular osmolarity occurs, resulting in damage to plasma membranes and organelles, as well as the freezing of extracellular water. During the thawing phase, the osmotic gradient is reversed, driving extracellular water into the cell causing swelling and leading to its rupture. Lastly, the damaging effects of cold intensify during the second freezing phase.

A study by Oliver et al.[8] reported on a cohort of patients who were not suitable for surgical treatment, that included 48 tumors across various molecular subtypes and an average diameter of 17 mm. They observed a 90% local disease control rate. In the light of these findings and the advantages of this technique, we aim to assess its efficacy in patients with indication for BCS [9, 10]. Initial results from the first 20 cases in this study were published in 2022 [11, 12].

In the present study, we performed pre-surgical cryoablation in 60 infiltrating ductal carcinomas (IDC) measuring up to 2 cm and analyzed the surgical specimens to evaluate the presence or absence of residual invasive cancer (RIC).

Two ongoing clinical trials, FROST (Freezing Instead of Removal Of Small Tumors) and ICE-3 (Cryoablation of Small, Low-Risk Breast Cancer), are examining cryoablation as a potential substitute for surgery, with encouraging preliminary results published in 2021 [13].

## Material and methods

This study was approved by the institutional review board and the hospital ethics committee. It was designed as a prospective observational study focused on the validation of diagnostic and interventional tests, registered under the code HULP: PI-4841 “Efficacy of cryoablation as a treatment for early and low-risk breast cancer”. The study was conducted with informed consent obtained from all patients and was not funded by any external grant or corporate entity.

### Study population

This single-center study included 59 participants with 60 infiltrating ductal carcinomas (IDC) ≤ 2 cm, recruited between March 2021 and June 2023. Participants were comprehensively informed by a department radiologist about the objectives of the study and the potential benefits of foregoing future surgery. The participants were women from the healthcare area served by La Paz Hospital in Madrid. All the participants were diagnosed with ER + /HER- breast cancer, luminal A or B ≤ 2 cm, and were consecutively referred from the breast pathology units of the hospital's gynecology or general surgery departments. All cases were presented to the multidisciplinary committee for BCS and selected to participate in the study.

Imaging assessments and cryoablation procedures were performed by one of seven senior breast radiologists (TDBD, MJRN, MVCCH, JMOG with over 20 years of experience; FGM, YNM, DGA with more than 5 years of expertise).

Diagnostic imaging tools included mammography and tomosynthesis with mammograph (Siemens Inspiration Prime). Core needle biopsies (CNB) were conducted under US guidance, using the 14G automatic system (Acecut de Léleman or Bip-Histocore). Axillary assessment was performed using ultrasound. All tumors were tagged with a ferromagnetic seed.

The inclusion criteria for the pre-surgical cryoablation study were as follows:

Patients aged 18 or older, suitable for BCS, with no requirement for primary systemic therapy, conclusive CNB of IDC ≤ 2 cm visible on ultrasound, ER + /HER2- status and radiologically confirmed negative axillary status.

Exclusion criteria were defined as the presence of an extensive intraductal component of the tumor, evidenced by visible microcalcifications on mammogram, or non-mass enhancements on magnetic resonance imaging (MRI) exceeding 2 cm. Participants with HER2 + luminal tumors or axillary involvement detected on ultrasound were also excluded from the study.

For patients with coexisting IDC/DCIS, MRI was advised for accurate staging, tumor size estimation and to exclude enhancements larger than 2 cm.

### Cryoablative procedure

A total of 60 cryoablations were performed in the ultrasound-guided breast interventional room within the hospital’s radiology department. The procedures were completed on an outpatient basis, with no need for subsequent inpatient monitoring.

Initial steps included local anesthesia infiltration followed by the placement of a ferromagnetic seed within the tumor, using either the 18G (Magseed Sysmex) or 14G (Sirius) coaxial, depending on availability.

Using the anesthesia entry point, the cryoprobe was introduced under ultrasound guidance using the (Siemens Acuson 2000). The cryoablation device used in the study was the (ICEfx Galil Boston Scientific) with Argon bottles supplied by the institution. The needles used were (IceSphere) 17G or (IcePearl) 14G. The choice of needle was determined by tumor size and the presence or not of an intraductal component associated with the infiltrating tumor.

The standardized triple-phase protocol (freeze-thaw-freeze) was applied as directed by the manufacturer, each segment lasting ten minutes. Monitoring by ultrasound was conducted to observe the growth of the ice ball in real time, ensuring the tumor was fully encased with margins of at least 1cm, and to assess the proximity of the ice front to the skin surface. **Video.**

In cases where tumors were situated superficially and the ice ball was approaching the skin surface, a warm saline solution bag was applied externally to prevent cold burns. The bag was placed on the breast skin, shielded with surgical gauze and copious ultrasound gel to avoid adhesions. Saline hydrodissection, as described by other authors, was not practiced [11, 12].

Post-cryoablation, a control mammogram was performed to verify the accurate positioning of the seed. Surgical interventions were scheduled based on operating room availability and clinical priority, with the time from cryoablation to surgery remaining unaffected.

Pathological evaluation of core biopsies and lumpectomy specimens was conducted at the hospital's pathology department in accordance with the latest guidelines from the Royal College of Pathologists of Australasia and was overseen by three expert breast tumor pathologists.

The margins of each specimen were marked with India ink to indicate the appropriate orientation of the piece and documented to establish the distance to each edge. The fixation of the specimen ranged from 12 to 72 h. At least one block from each surgical margin as well as the entire tumor or tumor bed, were included.

### Statistical analysis

Key variables considered in this study include:

Residual Invasive Cancer, Histological Grade, PR, Ki67, DCIS, Subtype, Time to Surgery, Seed, Needle, Distance to Skin and MRI.

The current sample size of 60 achieved a power of 0.62. To attain a power of 0.80, a sample size of 91 would be necessary. The recruitment of a larger cohort is ongoing.

Initial analysis involved Fisher’s exact test to identify any significant association between “Residual Invasive Cancer” and other categorical variables. For continuous covariates, univariable logistic regression models were used. Through this approach, covariates such as DCIS, Seed, Size, and Time to Surgery were shortlisted for a multivariable model, each exhibiting a univariable test p-value of < 0.25.

Subsequent logistic regression models were evaluated, considering potential confounding factors, and the optimal model with the best fit was proposed for bivariable logistic regression.

## Results

A total 70 participants with 71 low-risk IDC ≤ 2 cm were referred from the hospital’s multidisciplinary breast pathology committee for presurgical cryoablation.

Eleven were excluded: three for triple-negative cancer, three due to size > 2 cm on MRI scans, and five declined to participate.

**Fig. 1:**
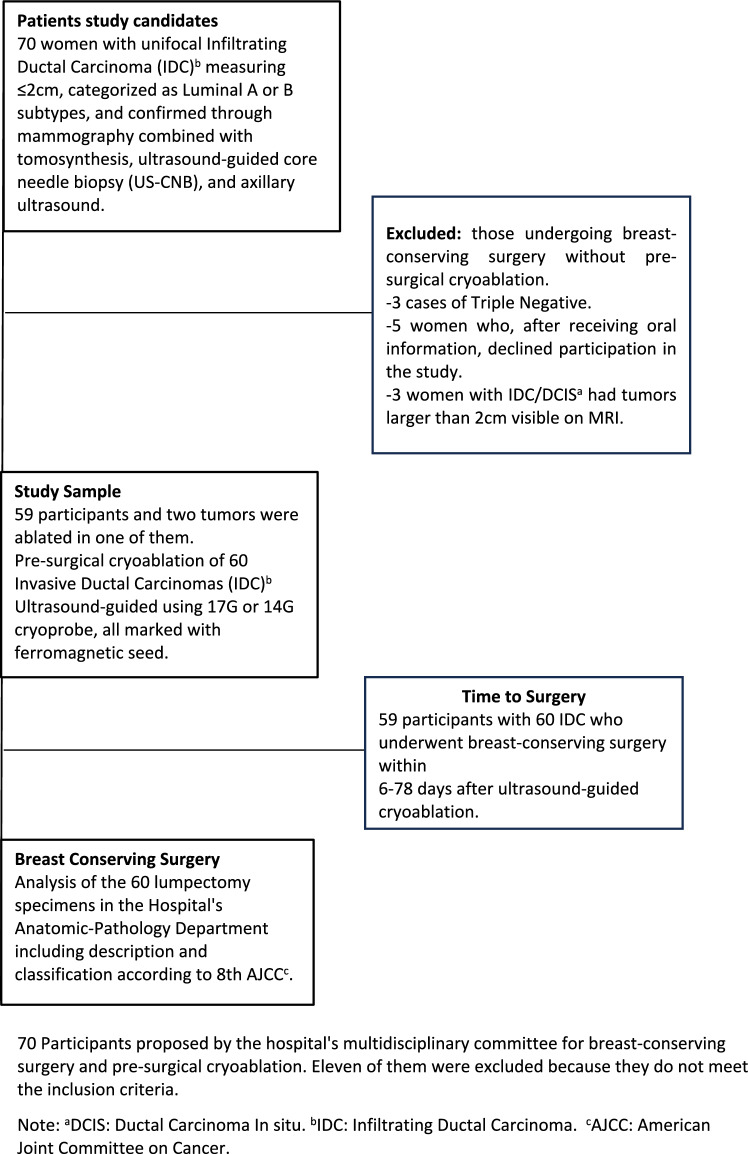
Flowchart. 70 Participants proposed by the hospital’s multidisciplinary committee for breast-conserving surgery and pre-surgical cryoablation. Eleven of them were excluded because they did not meet the inclusion criteria. Note: ^a^DCIS: Ductal Carcinoma In situ. ^b^IDC: Infiltrating Ductal Carcinoma. ^c^AJCC: American Joint Committee on Cancer

Preoperative ablation was performed on 59 women, accounting for 60 low-risk IDC ≤ 2 cm (Fig. 1).

The mean age of participants was 63 ± 8 years.

Table 1 summary of Cryoablated Breast Tumors ≤ 2 cm, details the molecular characteristics of the tumors as well as their size, stage, type of seed, needle type, distance to the skin surface, adverse effects and time from cryoablation to surgery. Among the tumors, 23 were G1 (38%) and 37 were G2 (62%); all were ER + /HER2-, while 7 (12%) were progesterone receptor-negative (PR-). Ki:67 average was 13 ± 7.2% (range 3–30%), with 34 classified as Luminal A and 26 as Luminal B. The average tumor size by ultrasound was 10 ± 4 mm.

**Table 1:** Summary of Cryoablated Breast Tumors ≤ 2 cm

Characteristics	IDC^a^
	n = 60
Age	63 ± 8
Range	31–81
Tumor grade	
Low (G1)	23 (38)
Moderate (G2)	37 (62)
HR status	
Estrogen Receptors positive (ER +)	60 (100)
Progesterone Receptors positive (PR +)	53 (88)
Her2 negative (Her2-)	60 (100)
Ki 67	
Mean ± SD	13 ± 7.2
Range	3–30
Subtype	
Luminal A	34 (57)
Luminal B	26 (43)
Tumor size by ultrasound (mm)	
Mean ± SD	10.1 ± 3.6
Range	4–20
Cancer stage	
T1a ≤ 5 mm	5 (8)
T1b > 5 mm–10 mm	35 (58)
T1c > 10 mm– ≤ 20 mm	20 (33)
Distance to skin surface (mm)	
Mean ± SD	9 ± 3.2
Range	2–18
Breast Laterality	
Right	32 (53)
Left	28 (47)
Screening program	30 (50)
Adverse effects post-procedure	
Mild	4 (7)
Time to surgery (days)	
Mean ± SD	21.8 ± 13.8
Range	(6–78)
Needle	
(IceSphere)^b^ 17G	40 (67)
(IcePearl)^c^ 14 G	20 (33)
Seed	
(Sysmex) 18G	45 (75)
(Sirius) 14G	15 (25)

Note: Unless otherwise specified, data are numbers of cancers with percentages in parentheses. IDC = Infiltrating ductal cancer. Data are mean ± standard deviation. Data in parentheses are percentages. (IceSphere)^b^ = 17G Needle. (IcePearl)^c^ = 14G Needle. Range: minimum and maximum value. Data in parentheses are percentages

**Fig. 2:**
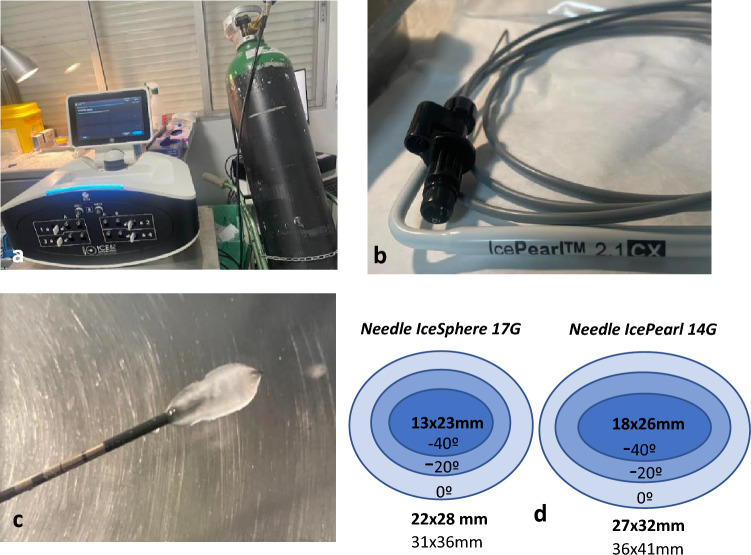
(**a**) Gas argon bottle and intuitive user interface with eight needle connection ports. (**b**) Cryoablation needle IcePearl 14G. (**c**) Iceball visible during the needle test. (**d**) Range of ablation zone sizes in ellipsoidal and spherical shapes

Forty patients underwent cryoablation using an (IceSphere) 17G needle, 20 using an (IcePearl) 14G cryoprobe. Forty-seven were marked using a (Sysmex) 18G ferromagnetic seed, while a (Sirius) 14G seed was used in 13 tumors (Fig. 2 a–d) Cryoablation Argon Gas System.

The average time between ablation and surgery was 22 days. Seventy-eight days was the longest a patient waited between procedures. This patient was initially deemed unsuitable for surgery due to comorbidities and was later reassessed and identified as a potential BSC candidate.

Results have been analyzed according to the type of tumor as either IDC Pure or IDC Mixed.

“Pure IDC” is defined as infiltrating ductal carcinoma in the absence of intraductal carcinoma, detailed as such by the pathologist after analyzing at least three cylinders obtained with 14G CNB for disease diagnosis.

“Mixed IDC” is defined as infiltrating ductal carcinoma (IDC) diagnosed after obtaining at least three tissue cylinders with a 14G CNB, with a pathology report consistent with the presence of ductal carcinoma in situ (DCIS) that includes a description of the grade, architectural pattern (solid, cribriform, papillary, or comedo), and the presence or absence of necrosis or microcalcifications (Table 2).

**Table 2:** Analysis of the post-cryoablation surgical specimen according to the result of the diagnostic CNB pre-ablation

Variables analyzed	RIC^a^	Absence residual IDC	Size (mm)	DCIS^d^ nests	Needle	Seed
IDC pure^b^ n = 38(63)	1(3)	37(97)	9.6 ± 3.7	4(10)	(IceSphere)^e^ 29(76)	(Sysmex)^g^ 30(79)
Range^i^			4–20		(IcePearl)^f^ 9(24)	(Sirius)^h^ 8(21)
IDC mixed^c^ n = 22(37)	4(18)	18(82)	11 ± 3.2	5(23)	(IceSphere)^e^ 11(50)	(Sysmex)^g^ 17(77)
Range^i^			5–19		(IcePearl)^f^ 11(50)	(Sirius)^h^ 5(23)

^a^RIC = Residual Invasive Cancer

^b^IDC pure = pathologist’s report does not reflect the presence of associated DCIS in the CNB cylinders

^c^IDC mixed = pathologist’s report reflects the presence of associated DCIS in the CNB

^d^DCIS = Ductal Carcinoma in situ

^e^Needle IceSphere = 17G

^f^IcePearl = 14G

^g^Introductor ferromagnetic seed (Sysmex) = 18G

^h^(Sirius) = 14G

^i^Range: minimum and maximum value. Data in parentheses are percentages

Of the 60 tumors ≤ 2 cm that underwent cryoablation:

Thirty-eight corresponded to IDC Pure with an average size of 9.6 ± 3.7 mm. Of these, 29 were treated with (IceSphere) and 9 with (IcePearl).

In 37 cases, Residual Invasive Cancer (RIC) was absent, and only one of them displayed intense post-cryoablation changes with isolated IDC nests measuring 5 mm overall. It was categorized as pT1aN0Mx according to the 8th AJCC pTNM classification. The remaining 37 showed intense post-treatment changes and were labeled pT0N0Mx.

**Fig. 3:**
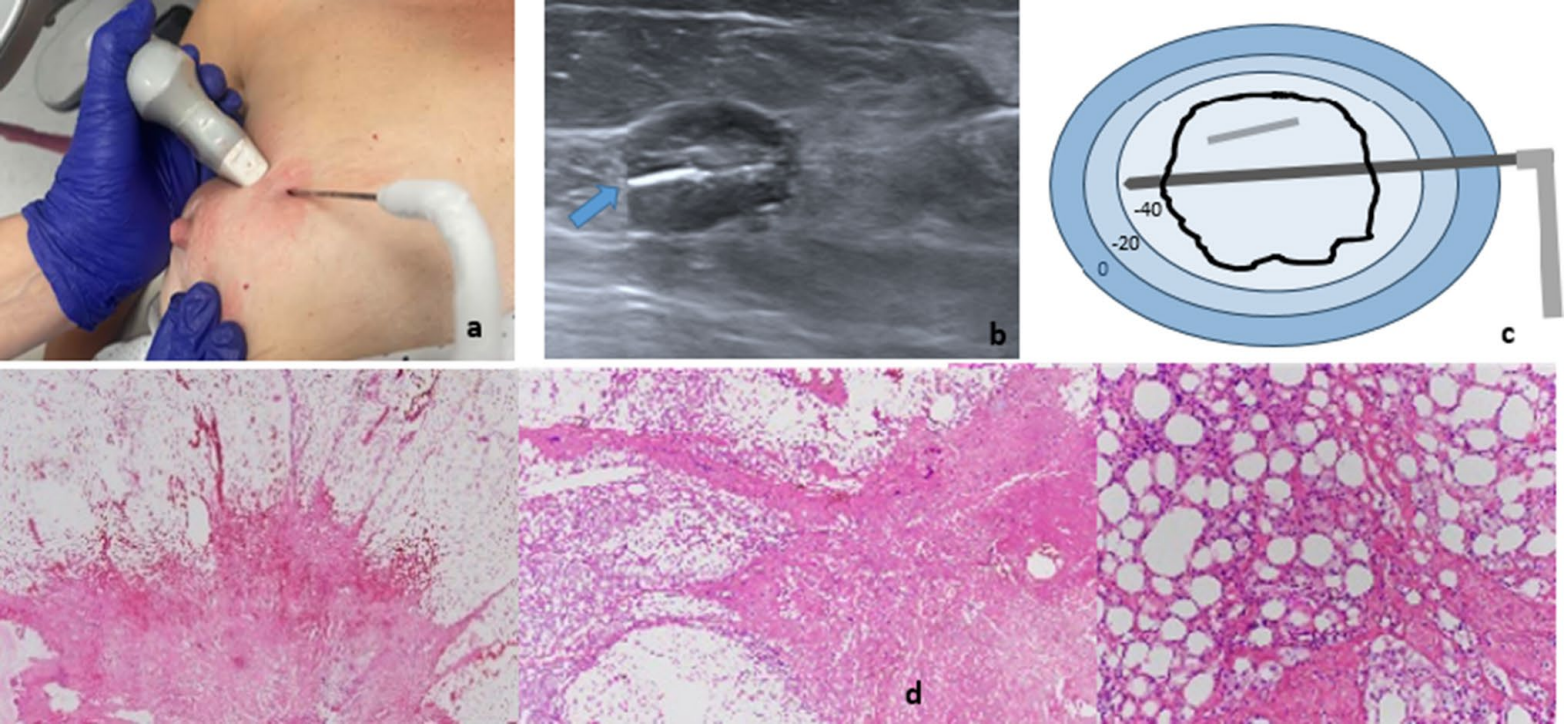
A 70-year-old female with pure invasive ductal carcinoma (IDC) measuring 19mm located in the upper inner quadrant of the right breast. (**a**) Percutaneous ultrasound-guided technique. (**b**) Irregular 19mm nodule with a Sirius seed located within the tumor (blue arrow). (**c**) Schematic representation of the procedure using the IcePearl needle. (**d**) Cicatricial fibrosis and hemorragic necrosis post-cryoablation. Absence of infiltrative component

In four cases, DCIS nests were reported away from the cryoablation area, indicating a 97% effectivity rate for pure IDC. Figure 3 (a–d) IDC Pure.

The only patient in which ablation was not successful did not have a preceding MRI, given the context of pure histology.

Twenty-two were IDC Mixed, with an average size of 11 ± 3.2 mm. Half were treated with (IceSphere) and half with (IcePearl). Four cases manifested intense post-treatment changes, revealing RIC foci between 1–4 mm, staged ypT1aN0Mx. Thus, the procedure’s efficacy for mixed IDC was 82%.

Out of the 22 IDC/DCIS cases, 18 underwent MRI and 4 declined the imaging procedure.

Out of the 4 unsuccessful procedures, MRI was not performed in two of them.

**Fig. 4:**
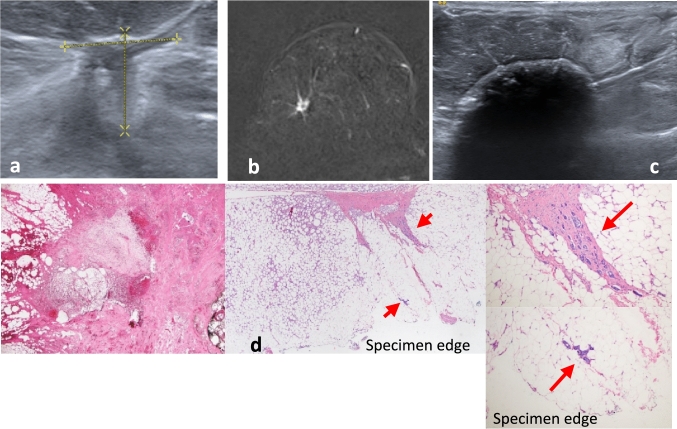
A 45-year-old woman diagnosed with invasive ductal carcinoma (IDC) in the right lower outer quadrant of the right breast. The figure illustrates the process of cryoablation and subsequent pathological analysis of the excised specimen. (**a**) A poorly demarcated nodule visible by US of 14 mm. (**b**) The MRI reveals a mass-like uptake with spiculated borders. (**c**) The Ice ball generated by IcePearl needle has engulfed the tumor. (**d**) Intense post cryoablation changes with a hemorrhagic central zone devoid of malignancy. Within the periphery zone of the steatonecrosis, nests of DCIS and several 2 mm foci of RIC are observed (arrows). Note: *RLO* right lower-outer quadrant, *RIC* residual invasive cancer, *DCIS* Ductal Carcinoma In situ

All specimens were reported as tumor-free margins. Five samples contained DCIS nests away from the tumor bed. Figure 4 (a–d) IDC Mixed.

In all cases, the marking seed was precisely located by the surgeon. Specimen X-ray was used for radiological confirmation of the location of the tumor and the adjacent seed.

Macroscopic analysis revealed a central violaceous hemorrhagic lesion, with a metallic marker (seed) surrounded by a prominent yellow halo, corresponding to steatonecrosis.

**Fig. 5:**
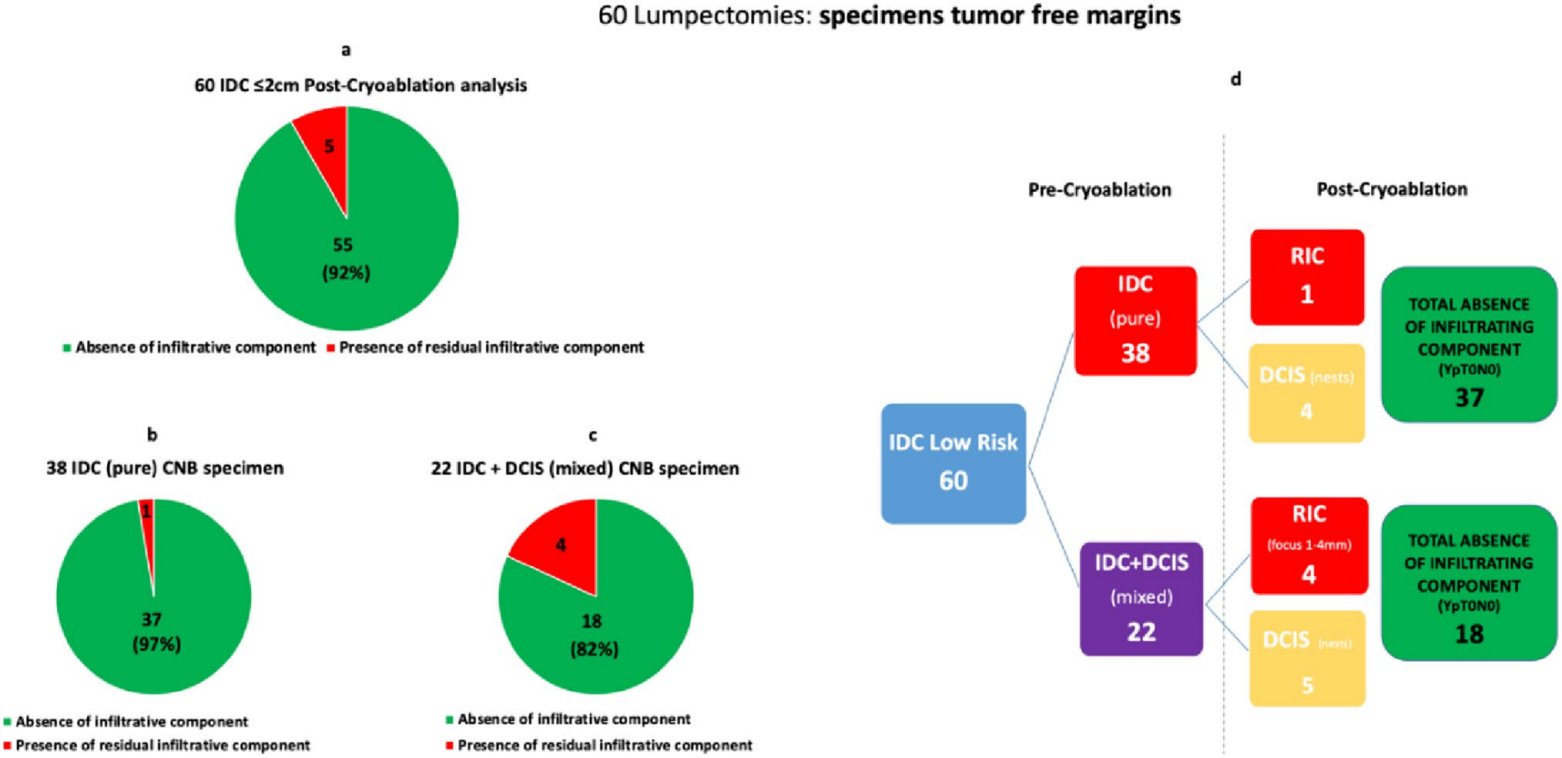
Analysis of Cryoablation’s Efficacy in 60 early and low-risk Breast Cancer. Results Diagram. (**a**) Global efficacy with five instances of failure. (**b**) Efficacy in pure IDC cases. Out of 38 cases, one resulted in failure. (**c**) Efficacy in mixed IDC cases. Out of 22 cases, four resulted in failure. (**d**) Among 38 pure IDC cases, an infiltrative component was absent in 37 cases, while residual invasive cancer (RIC) was present in 1 case. Among 22 mixed IDC cases, absence of residual infiltrative component was observed in 18 cases, and RIC was present in 4 cases

Figure 5 (a–d) Analysis of Cryoablation’s Efficacy in 60 early and low-risk Breast Cancer. Results Diagram.

### Statistical analysis

The results indicate that there is a significant relationship between the variables DCIS and Size, with the presence of Residual Invasive Cancer (RIC) (p = 0.09, CI 95%), with a positive odds ratio. A positive DCIS and a larger tumor size increase the probability for RIC, with an odds ratio (OR) for DCIS of 7.43 and an OR for Size of 1.27 (Table 3).

**Table 3:** Statistical analysis of variables related to RIC

Variable name (dichotomic)	Fisher’s exact test p-value
Histological grade	1
PR	0.475
DCIS	0.056
Subtype	0.640
Seed	0.094
Needle	0.322
MRI	0.648
Variable name (continuous)	Univariable logistic regression models. Variable p-value
Size	0.061
Ki:67	0.799
Time to surgery	0.063
Distance to skin	0.208

Residual Invasive Cancer	Odds ratio	Std. err	z	P >|z|	[95% conf. interval]
1.DCIS	7.4372	8.875787	1.68	0.093	0.7170658 77.1365
Size	1.274562	0.1835949	1.68	0.092	0.9610567 1.690334
_cons	0.0017177	0.0037232	−2.94	0.003	0.0000254 0.1202059

Results of Fisher’s exact test (dichotomic variables) and Univariate Logistic Regression (continuous variables). Results of the best fitted Bivariable Logistic Regression Models N = 60

Significant association p value < 0.25

Number of obs = 60

LR chi2(2) = 7.30

Prob > Chi2 = 0.0260

Log likelihood = −13.560805

Pseudo R2 = 0.2120

Other variables, including PR- status, time to surgery, seed type, histological grade or subtype, did not correlate with the presence of RIC in the lumpectomy specimen.

## Discussion

Breast cancer is the most commonly diagnosed cancer in the world, with a higher incidence in developed countries. Early diagnosis rates are high, ensuring that numerous small tumors are surgically treatable [14, 15].

Currently, the trend is to have entered a phase of de-escalation in treatment. In our study, cryoablation effectively eradicated 97% of pure IDC and 82% of mixed IDC cases. Among the variables examined, both DCIS and tumor size show significant positive correlation with RIC (p = 0.09, 95% CI). Positive DCIS significantly increases the likelihood of finding Residual Invasive Cancer within the surgical specimen post-cryoablation, with an OR of 7.43. Similarly, with every unit increase (mm) in tumor Size, the OR for RIC increases 1.2. These findings are in concordance with those published by Poplack et al. [16].

The presence of DCIS nests away from the cryoablation focus do not impact patient management or prognosis, given that all lumpectomy specimens had clear surgical margins.

Studies have shown that unifocal T1-T2 tumors may show subclinical invasive carcinoma foci or DCIS foci remote to the primary tumor. Holland published an examination of mastectomy specimens from patients diagnosed with IDC, revealing that 20% of invasive T1-T2 cancers had foci within 2cm of the primary tumor, and 43% extended beyond this boundary [17].

In 2013 a consensus document elaborated by a multidisciplinary expert panel was released, indicating that if an infiltrating cancer is completely surgically removed (tumor-free margins confirmed by pathology), there is no increased relapse risk if patients complete subsequent adjuvant treatments like radiotherapy (RT) and endocrine therapy [2, 18]. Outside cryoablation areas, DCIS nests will likely disappear after adjuvant RT [19].

Clinicopathologic feature analysis between IDC and IDC/DCIS patients reveals that mixed tumors display over 50% multifocality compared to pure tumors. Additionally, FDG PET analysis suggests that unifocal IDC/DCIS tumors may present with undetected radiological multifocality [20].

The impact of various factors on the elevated re-excision rate following breast-conserving surgery (BCS) has also been examined. A study emphasized the crucial role of coexisting DCIS in producing positive margins, as delineating these tumors may be challenging [21]. Accurate estimation is vital for cryoprobe selection. Substantial underestimation by US is recognized in invasive lobular carcinoma (ILC) and IDC/DCIS [22, 23]. Some argue that MRI is the best predictor for tumor size, especially for mixed tumors. The presence of DCIS reduces lesion visibility and margin definition [24, 25]. Thus, for mixed infiltrating tumors, the ice ball generated during cryoablation should widely encompass the tumor size estimated by both ultrasound and MRI.

While acknowledging that 60 cases may not constitute a large sample, in cases 9 and 14 in our series RIC was present in the surgical specimen, and both were IDC/DCIS tumors of 10 and 12 mm treated with IceSphere needles. Following these cases, we opted to choose probes that would allow for the formation of larger ice balls. In the subsequent 3 patients with mixed tumors measuring 10 and 12 mm treated with 14G IcePearl probes, the technique was successful.

Ideally, as depicted by the needle manufacturer, completely encasing the tumor with a 5-10 mm margin in its major diameter is optimal. At 0 °C, the ice is visible but may not be lethal. Lethal temperatures are reached at -20° and −40 °C. A larger tumor, of up to 12 mm, may be encased with sufficient 5 mm margins using an IceSphere needle, as it may achieve a lethal area of approximately 22 mm × 28 mm.

However, when dealing with IDC/DCIS tumors, where establishing tumor margins and size can be more challenging, using an IcePearl needle will increase the likelihood of treatment success as we can ensure a maximum lethal zone of 27 mm × 32 mm.

Based on our experience, a 17G cryoprobe is suitable for tumors ≤ 12 mm without DCIS in the CNB and ≤ 10 mm if the tumor shows an intraductal component.

In 2004, Sabel et al. [26] reported a 100% success rate with argon cryoablation for tumors under 1cm and 82% for those under 2 cm, after studying 27 tumor samples.

In two studies on breast tumor cryoablation published in 2015 and 2016, success rates of 92–95% were reported if DCIS nests away from the ablated area were not considered as failures [16, 22].

**Fig. 6:**
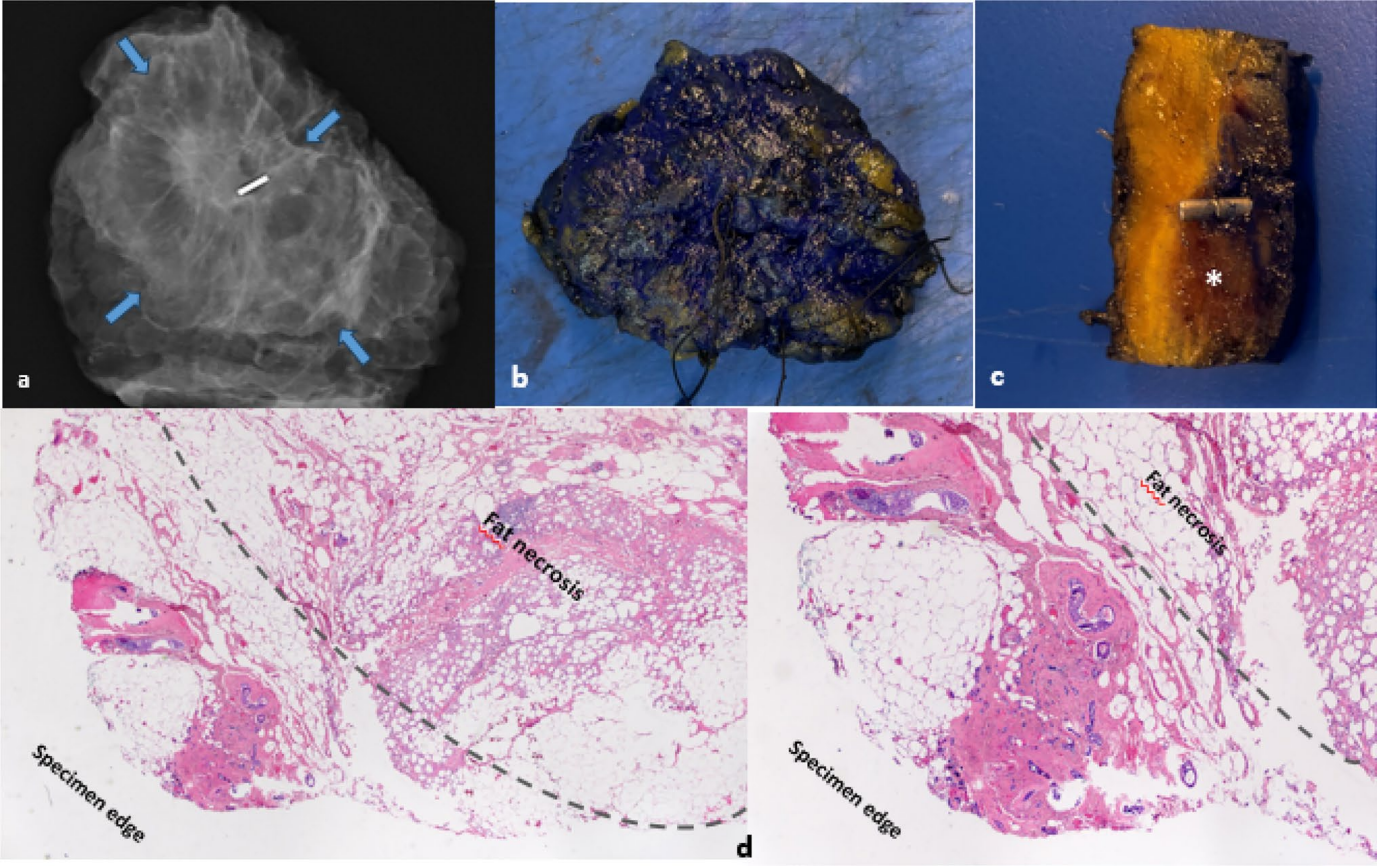
Macroscopic and microscopic images of the surgical specimen with 14G seed after cryoablation of a 12 mm HR + Her2- Ki 67:10 Luminal A IDC/DCIS tumor. (**a**) Radiographic representation of the surgical specimen revealing the coexistence of the tumor, the seed, and a peripheral region displaying post-cryoablation fat necrosis (indicated by blue arrows). (**b**) Macroscopic view of the specimen marked with ink. (**c**) One of the serial fillets through the center of the hemorrhagic necrosis * with (Sirius) seed. (**d**) Two-millimeter foci of Residual Invasive Cancer (RIC) distant from the region of fat necrosis marked by the dashed line and in proximity to the specimen’s periphery, maintaining separation from the inked margin. ypT1aN0Mx. Note: *IDC* Infiltrating ductal carcinoma, *DCIS* Ductal carcinoma in situ, *RIC* Residual invasive cancer

Our findings align with the data published by Poplack et al. [16] who found instances of residual infiltrating components in tumors with non-calcified DCIS alongside IDC. Similarly, our pathologists described millimetric RIC foci in the periphery of the specimens, not at the cryoablation bed center. We believe the technique becomes ineffective when tumors surpass ice ball dimensions. Figure 6 (a-d) Macroscopic and microscopic images of the surgical specimen.

Six patients reported mild discomfort that did not warrant intervention, and only one experienced moderate to severe pain that responded well to standard oral analgesia and anti-inflammatory medication. Only one patient presented with a small (5 mm) skin vesicle.

Four out of the 5 cases where RIC foci remained post-procedure were associated with tumors containing DCIS. Both variables, DCIS and tumor size, were significantly associated with technique failure (p = 0.09).

This outcome is consistent with expectations, as tumors coexisting with DCIS are known to display greater multifocality that may not be detectable by imaging.

At our facility, MRI is used to stage luminal tumors under 2 cm that show an intraductal component in the CNB, which is not apparent from microcalcifications on mammography.

Among 22 patients with mixed IDC/DCIS, 4 declined MRI. Subsequent specimen assessment revealed RIC in 2 of these cases (50%). Thus, there is a possibility that tumor size was underestimated in these cases.

The first case of technique failure involved a mixed tumor where the participant declined MRI staging. The tumor was initially assessed ultrasonographically at 12 mm. However, an X-ray of the surgical specimen revealed spicules with pathological microcalcifications extending up to a 2 cm diameter. Additionally, the pathologist described an indurated tumor within the specimen, with a maximum diameter of 16 mm. This patient was number 12 in our series, and due to our limited experience at that time, we chose a 17G needle which was not appropriate for the actual size of the tumor.

In the only case with a 16 mm pure IDC that showed post-ablation RIC, a 17G probe was used due to the unavailability of a 14G needle, leading to insufficient ice coverage of the tumor.

The conclusion derived from our study is that the technique becomes ineffective when the tumor exceeds the size of the surrounding ice area. It is crucial to make the most accurate estimation possible of tumor size, relying on all available imaging methods.

Efficacy is related to the chosen ice ball diameters, proper probe placement, and the exact tumor size.

Our study had some limitations, including being single institution, a potentially insufficient sample size and the lack of universal MRI staging.

Pending the results of studies like ICE3 and others, coupled with advancements in axillary surgery de-escalation like the omission of SLNB based on the conclusions of the St Gallen 2023 SOUND study, cryoablation could emerge as a viable treatment option for pure IDC ≤ 2 cm [13, 27–29].

With consensus from a multidisciplinary medical board, physicians with experience in breast pathology are key in the selection, procedure execution and follow up of these patients.

### Supplementary Information

Below is the link to the electronic supplementary material.

Supplementary file1 (MP4 51174 KB)

## Abbreviations

ER +: Estrogen Receptor positive
IDC: Infiltrating ductal carcinoma
DCIS: Ductal carcinoma in situ
RIC: Residual Invasive cancer
CNB: Core needle biopsy
TTS: Time to surgery
DTS: Distance to Skin
PR-: Progesterone Receptor negative
BCS: Breast Conserving Surgery
ypT0: Total absence of invasive component according to the 8th edition of the American Joint Committee on Cancer (AJCC) staging system
